# Improved analgesia and reduced post-operative nausea and vomiting after implementation of an enhanced recovery after surgery (ERAS) pathway for total mastectomy

**DOI:** 10.1186/s12871-018-0505-9

**Published:** 2018-04-16

**Authors:** Catherine Chiu, Pedram Aleshi, Laura J. Esserman, Christina Inglis-Arkell, Edward Yap, Elizabeth L. Whitlock, Monica W. Harbell

**Affiliations:** 1 Department of Anesthesia and Perioperative Care, University of California, San Francisco, 513 Parnassus Ave, S436, Box 0427, San Francisco, CA 94143 USA; 2 Department of Surgery, University of California, San Francisco, San Francisco, CA USA

**Keywords:** Fast-track surgery, ERAS pathway, Total mastectomy, Breast surgery, Pec blocks

## Abstract

**Background:**

Enhanced Recovery After Surgery (ERAS) pathways have been shown in multiple surgical disciplines to improve outcomes, including reduced opioid consumption, length of stay, and post-operative nausea and vomiting (PONV). However, very few studies describe the application of ERAS to breast surgery and even fewer describe ERAS for outpatient surgery. We describe the implementation and efficacy of an Enhanced Recovery After Surgery (ERAS) pathway for total skin-sparing mastectomy with immediate reconstruction in an outpatient setting.

**Methods:**

We implemented an evidence-based, multimodal ERAS pathway for all patients undergoing total skin-sparing mastectomy surgery with immediate reconstruction at a single 23-h stay surgery center. Highlights of the ERAS pathway included: preoperative acetaminophen, gabapentin, and scopolamine; regional anesthesia for the breast (Pectoral blocks type 1 and 2 or paravertebral block); and intraoperative dexamethasone and ondansetron. This retrospective study included all American Society of Anesthesiology (ASA) Class 1–3 patients undergoing total skin-sparing mastectomy surgery with immediate reconstruction between July 2013 and April 2016. We compared 96 patients who were in the ERAS pathway (ERAS group) to a retrospective cohort of 276 patients (Pre group). The primary outcome was total perioperative opioid consumption. Secondary outcomes were highest postoperative pain scores, incidence of PONV, and length of stay.

**Results:**

Patients in the ERAS group had significantly lower total perioperative opioid consumption compared to the Pre group (mean (SD): 111.4 mg (46.0) vs. 163.8 mg (73.2) oral morphine equivalents, *p* < 0.001). Patients in the ERAS group also had a lower incidence of PONV (28% vs. 50%, *p* < 0.001). Patients in the ERAS group reported less pain in the recovery room, with a two-point decrease in highest pain score (median [interquartile range (IQR)]: 4 [2,6] in ERAS group vs. 6 [4,7] in Pre group, *p* < 0.001). There was no clinically significant difference in length of stay (median [IQR]: 1144 min [992, 1259] in ERAS group vs. 1188 [1058, 1344] in Pre group, *p* = 0.006).

**Conclusion:**

Implementation of an ERAS pathway for total skin-sparing mastectomy with reconstruction that incorporates regional anesthesia is feasible in a 23-h-stay hospital. Patients in the ERAS pathway had improved post-operative analgesia and reduced post-operative nausea and vomiting.

**Electronic supplementary material:**

The online version of this article (10.1186/s12871-018-0505-9) contains supplementary material, which is available to authorized users.

## Background

Breast cancer is the most common cancer diagnosis in women, with 30–40% of patients undergoing mastectomy as treatment [1]. As 20–60% of mastectomy patients develop chronic pain [2, 3], there is increasing attention to improving acute pain control as a potential means of preventing chronic postsurgical pain [4, 5]. Poor pain control postoperatively has also been associated with worse quality of life outcomes, including impaired sleep and physical function in the postoperative period and is one of the most common reasons for postsurgical hospital readmission, which can contribute to health care costs [5]. Further, given the opioid epidemic in the U.S., there is a nationwide movement to reduce opioid administration perioperatively [6, 7].

Enhanced recovery after surgery (ERAS) pathways for various surgery types have successfully implemented evidence-based practices that improve patient outcomes, including reduced opioid consumption, decreased post-operative nausea and vomiting (PONV), and decreased hospital length of stay [8–11]. While there is a plethora of data supporting ERAS pathways for other types of surgery, there is limited data on the application of ERAS principles to breast surgery and very few studies that examine the implementation of ERAS in an outpatient setting [12–14]. Of the ERAS studies set in the outpatient setting, all are proof-of-concept studies with small sample sizes and restrictive patient inclusion criteria. “Efficacy” trials such as these provide evidence that the approach may be successful under ideal conditions, but real-world applicability of the concepts to a clinically heterogeneous population – is not known.

We describe the implementation of a multimodal, ERAS pathway for all patients undergoing total skin-sparing mastectomy with immediate reconstruction in a 23-h stay hospital, which was designed to improve postoperative pain and PONV. As a quality improvement initiative, our goal was to evaluate if the implementation of an ERAS pathway for mastectomy was associated with improved postoperative pain control and reduced incidence of PONV. This retrospective study was registered at Clinicaltrials.gov, identifier: NCT03181139.

## Methods

After obtaining Institutional Review Board approval at the University of California – San Francisco (UCSF) (Study Number 15-15907), we performed a retrospective chart review of all patients who underwent total skin-sparing mastectomy with immediate reconstruction at UCSF Mount Zion Hospital between July 1, 2013, and April 30, 2016. To evaluate the efficacy of the ERAS pathway, we compared the first 96 patients who underwent the pathway (ERAS group) to the 276 patients immediately prior (Pre group) to implementation of the ERAS pathway on July 1, 2015. A post-hoc power calculation based on 250 patients in the Pre group and a minimum clinically important decrease of 30 mg oral morphine equivalents in the ERAS group determined a minimum sample size of 52 post-ERAS patients to achieve greater than 80% power to detect a difference in our primary outcome at an alpha = 0.05. Patients were identified for inclusion using surgical procedure codes specific for total mastectomy and included those having concurrent axillary dissection or lymphadenectomy, as well as immediate breast reconstruction with tissue expanders. Exclusion criteria included patients undergoing concurrent bilateral salpingo-oophorectomy, pre-pectoral placement of tissue expanders, or any flap reconstruction, such as deep inferior epigastric perforator, transverse rectus abdominus myocutaneous, or gracilis flaps, and previous inclusion in either the Pre or ERAS groups.

### Enhanced recovery after surgery (ERAS) pathway

**Fig. 1:**
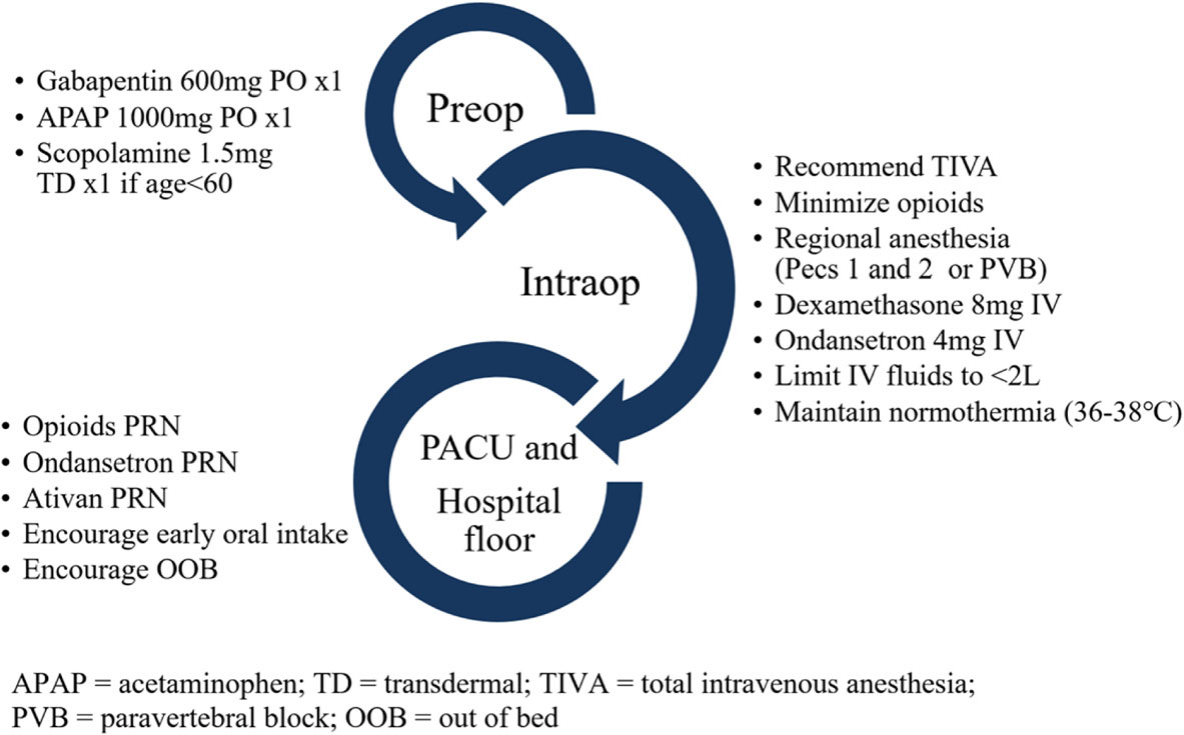
Highlights of the Enhanced Recovery after Surgery Pathway

The ERAS pathway was developed by a multidisciplinary team, addressing the needs of the patient in the preoperative, intraoperative and postoperative periods. Anesthesia, surgery, and nursing teams all received education on the proposed ERAS pathway. The ERAS pathway was implemented on July 1, 2015, in Mount Zion Hospital. Of note, this hospital transitioned from a tertiary care hospital supporting ICU level care to a 23-h stay hospital on February 1, 2015. All patients undergoing total mastectomy after implementation were included in the ERAS pathway (Fig. 1).

In the preoperative period, patients received educational materials about what to expect with their care, as well as detailed information about their multimodal pain regimen, including Pecs blocks. Preoperative interventions also included administration of oral acetaminophen 1000 mg and oral gabapentin 600 mg, and avoidance of prolonged fasting (i.e. clear liquids allowed up to 2 h prior to surgery). Patients younger than the age of 60 years, with a history of PONV also received a 1.5 mg transdermal scopolamine patch.

Intraoperative interventions included regional anesthesia (Pecs blocks or paravertebral block), total intravenous anesthesia (TIVA), intravenous dexamethasone 8 mg at the beginning of the case, intravenous ondansetron 4 mg prior to end of case, and minimal use of opioids. TIVA was defined as the avoidance of volatile anesthetics and nitrous oxide and was standardized as a propofol infusion with intravenous fentanyl and hydromorphone given at the anesthesia provider’s discretion with a recommendation to minimize opioids. Regional anesthesia, particularly Pecs blocks, were strongly encouraged in the ERAS pathway, however, the decision to perform Pecs vs. paravertebral blocks was left to provider preference. Pecs blocks Type 1 and 2 were performed after induction as described by Blanco et al. [15, 16] using a total of 60 mL of ropivacaine 0.2% for bilateral blocks and 30 mL of bupivacaine 0.25% for unilateral blocks. Paravertebral blocks were performed preoperatively as a single injection at T4 using 20 mL of ropivacaine 0.2% for each side of the block. The ERAS principles of maintenance of euvolemia and normothermia were emphasized in the perioperatively period. Fluids were limited to less than 2 l of isotonic crystalloids. Forced air warming perioperatively was encouraged for prevention of heat loss and temperatures less than 36 °C.

Postoperatively, patients received ibuprofen as needed for mild pain, acetaminophen combined with either hydrocodone or oxycodone as needed for moderate pain, and IV hydromorphone for severe pain unrelieved by oral pain medications, as assessed by the nurse caring for the patient. Lorazepam was offered to patients, as needed for muscle spasm in the PACU and hospital floor settings. Early mobilization and early oral intake were emphasized after surgery.

### Paravertebral block technique

Paravertebral blocks were placed preoperatively, and patients could receive 1-2 mg midazolam IV and/or fentanyl 50-100mcg IV for sedation for the block. For the paravertebral blocks, a parasagittal real-time ultrasound guided technique was employed using a 10–12 mHz linear transducer (Sparq Ultrasound System, Philips Ultrasound, Bothell, WA, USA). Patients were placed in either a sitting or prone position depending on provider preference. The skin was cleansed using a 2% chlorhexidine gluconate, 70% isopropyl alcohol solution, and aseptic technique was used throughout the procedure. The skin entry site was anesthetized with lidocaine 2% injected subcutaneously using a 27-gauge needle. A 21-gauge 10 cm Pajunk needle (SonoPlex, Stim cannula, Pajunk, Geisingen, Germany) was inserted caudad and in-plane to the ultrasound probe in a parasagittal oblique orientation at the T4 level. After negative aspiration, 1 mL of normal saline 0.9% was injected to confirm pleural depression. Then, ropivacaine 0.2% was injected in incremental doses with patients receiving 30 mL if unilateral or 60 mL if bilateral mastectomy.

### Pectoral blocks types I and II (Pecs blocks)

The Pecs blocks were performed in patients using a modification of the technique described by R. Blanco et al. [15, 16]. These blocks were placed post-induction but prior to incision. Patients were placed in the supine position with their arms abducted. A 10–12 MHz linear transducer (Sparq Ultrasound System, Philips Ultrasound, Bothell, WA, USA) was applied immediately medial to the coracoid process, underneath the clavicle in the parasagittal plane and redirected medially to obtain a view of the 2nd rib. The probe was then moved inferiorly and laterally down to the 3rd or 4th rib for identification of the pectoralis major, pectoralis minor, and serratus anterior muscle planes. A 22-gauge 5 cm Pajunk needle (SonoPlex Stim cannula, Pajunk, Geisingen, Germany) was inserted in a medial-to-lateral orientation with an in-plane approach, and 20 mL of local anesthetic was injected between the serratus anterior and pec minor. The needle was then withdrawn and 10 mL of local anesthetic was deposited between the pectoralis major and minor muscles. Ropivacaine 0.2% was used for majority of patients having bilateral Pecs blocks while bupivacaine 0.25% was used for majority of those with unilateral Pecs blocks.

### Data collection

The electronic health record (EHR) was reviewed for demographic characteristics (age, Body Mass Index (BMI), ASA class, type of surgery, history of PONV, smoking status, etc.), medication administration, and numerical rating scale (NRS) pain scores. The primary outcome was total perioperative opioid consumption. Secondary outcomes included highest NRS postoperative pain score, incidence of PONV, use of lorazepam, non-procedural operating room time, and length of stay.

The primary outcome of total perioperative opioid consumption was defined as the sum of the opioids administered to the patient in the preoperative area, operating room, post-anesthesia care unit (PACU), and on the hospital floor prior to discharge from the hospital. Total opioid consumption was converted to total oral morphine equivalents using the following formula: IV fentanyl (mcg) *0.3 + IV dilaudid (mg) *20 + IV morphine (mg) *3 + [# of oral hydrocodone 5 mg/acetaminophen 325 mg tablets * 5] + [# of oral oxycodone 5 mg/acetaminophen 325 mg tabs * 7.5] + [oral oxycodone (mg) *1.5] + oral morphine (mg). This equation is based on our institution’s opioid equivalence table which was developed from primary literature and is included in the Additional file 1.

The highest NRS pain score was recorded on a scale of zero (no pain) to ten (worst imaginable pain) and was assessed by nurses during the PACU and on the hospital floor as part of routine care. Incidence of PONV was defined as the use of any antiemetic, including ondansetron, prochlorperazine, and metoclopramide, from the time that patient left the operating room to the time of discharge. Lorazepam consumption in the PACU and on the hospital floor was extracted from the medication administration record in the EHR. Non-procedural operating room time was defined as the duration of time the patient was in the operating room (time from patient entering the OR to time patient leaves the OR) minus the surgery duration. During this time period, the patient was transferred from the gurney to the operating room table, underwent induction of general anesthesia, performance of the Pecs block, emergence from anesthesia, extubation and transfer from the operating room table to the gurney and ultimately to the PACU. PACU length of stay was defined as the time between patient PACU arrival and discharge to the floor. Hospital length of stay was defined as time out of PACU to time of hospital discharge.

Compliance of the pathway was also assessed using data extracted from the medication administration record, including dexamethasone, ondansetron, gabapentin and acetaminophen, scopolamine dose and timing. Compliance with TIVA was defined as the complete avoidance of volatile anesthetics and nitrous oxide and the use of propofol infusion for maintenance of anesthesia. Compliance with regional anesthesia was defined as the patient receiving either Pecs block or paravertebral blocks.

Data was analyzed with an “intention-to-treat,” such that patients who had surgery after ERAS implementation were considered part of the ERAS group even if not all components of the ERAS pathway were administered.

### Statistics

Data were reported as mean (standard deviation) for normally distributed continuous variables, as median (interquartile ranges (IQR)) for non-normally distributed continuous variables, and as count (percentage) for binary variables. *P*-values were calculated by a student’s t-test for parametric continuous variables, Mann-Whitney test for nonparametric continuous variables, and chi-square or Fisher’s exact test for categorical variables as appropriate. *P*-values < 0.05 were considered significant.

We performed a multivariable linear regression for both total opioid consumption and hospital length of stay, adjusting for age, prior opioid use, surgeon, and ERAS inclusion. Because the data for length of hospital stay was not normally distributed, a transformation using 1/(hospital length of stay)^2^ was performed prior to linear regression. However, as the conclusions were not different between the transformed and untransformed models of linear regression, we report the untransformed model for ease of interpretability. Post hoc sensitivity analyses were performed to assess the effect of the transition to a 23-h stay hospital on these outcomes.

Analyses were performed with R, version 3.3.2 (R Foundation for Statistical Computing) and Stata, version 13.1 (StataCorp LP) statistical packages.

## Results

### Patient characteristics

**Fig. 2:**
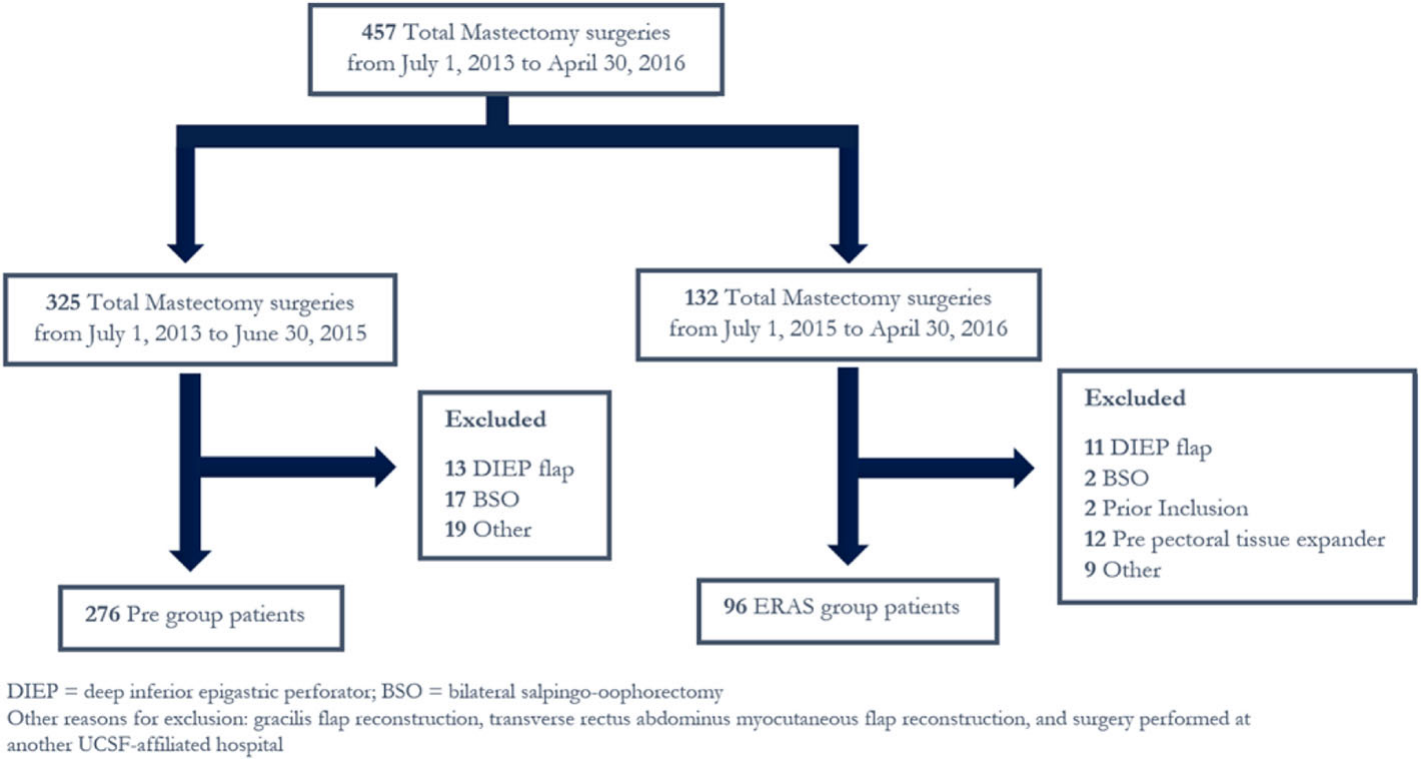
Patient Flow Diagram

A total of 457 patients underwent total skin-sparing mastectomy with reconstruction during the study period, and 372 patients fit the inclusion criteria (Fig. 2). Of these, 276 patients underwent surgery before implementation of the ERAS pathway (Pre group) and 96 patients underwent surgery after implementation of the ERAS pathway (ERAS group). Table 1 depicts demographic information of patients before and after implementation of the ERAS pathway. There were no significant differences found between the baseline characteristics and surgical data of the two groups, except for a slightly higher proportion of current smokers in the Pre group (5% in the Pre group vs 0% in the ERAS group, *p* = 0.03). There was a small difference in the surgeons performing the procedure with the proportion performed by Surgeon 3 decreasing from 51% to 38% and the proportion performed by Surgeon 4 increasing from 7% to 25%; however, surgical technique and approach was uniform amongst all four surgeons.

**Table 1:** Patient Demographics

Variable	Pre Group (*n* = 276)	ERAS group (*n* = 96)	*p*-value
Age (years), *mean (SD)*	48.8 ± 11.1	46.9 ± 8.91	0.24^*^
BMI (kg/m^2^), *mean (SD*)	24.5 ± 5.58	23.6 ± 4.20	0.32^*^
ASA class, *median*	2	2	0.60^*^
Current Smoker, *n (%)*	14 (5%)	0 (0%)	0.03^ψ^
Prior Smoker, *n (%)*	76 (30%)	21 (22%)	0.34^+^
Prior Opioid use, *n (%)*	29 (11%)	12 (13%)	0.85^ψ^
PONV History, *n (%)*	67 (24%)	22 (23%)	0.94^+^
Surgeon, *n (%)*			
1	1: 84 (30%)	1: 25 (26%)	
2	2: 31 (11%)	2: 11 (11%)	< 0.001^ψ^
3	3: 141 (51%)	3: 36 (38%)	
4	4: 20 (7%)	4: 24 (25%)	
Bilateral Mastectomy, *n (%)*	165 (60%)	56 (58%)	0.89^+^
Axillary Dissection / Lymphadenectomy, *n (%)*	204 (74%)	67 (70%)	0.51^+^

^+^*p*-values calculated from chi-square test

^*^p-values calculated from Mann-Whitney test

^ψ^*p*-values calculated from Fisher’s exact test

*BMI* body mass index, *PONV* post-operative nausea and vomiting

### Compliance with ERAS pathway

Overall, there was excellent compliance with the ERAS pathway. After implementation of the ERAS pathway, use of preoperative acetaminophen increased from 17% to 89%, gabapentin from 13% to 89%, scopolamine patch from 22% to 76% (*p* < 0.001). The use of regional anesthesia increased from 18% to 88% (*p* < 0.001). Of the patients who received regional anesthesia as part of their anesthetic, a majority of the Pre group received paravertebral blocks, whereas in the ERAS group, the majority received Pecs blocks. Although general anesthesia with total intravenous anesthesia was recommended as part of the pathway, there was only a modest increase in TIVA from 8% to 33% (*p* < 0.001). A majority of patients in the ERAS group received dexamethasone 8 mg at the beginning of the operation (from 18% to 53%), and there was no change in ondansetron administration near the end of the operation (*p* = 0.11). Further details with pathway utilization and compliance can be found in Table 2.

**Table 2:** Pathway utilization and compliance

Variable	Pre Group (*n* = 276)	ERAS Group (*n* = 96)	*p*-value
Preoperative Acetaminophen, *n (%)*	48 (17%)	86 (89%)	< 0.001^+^
Preoperative Gabapentin, *n (%)*	35 (13%)	86 (89%)	< 0.001^+^
Scopolamine patch, n (%)	62 (22%)	73 (76%)	< 0.001^+^
Nerve Block, *n (%)*	51 (18%) PVB: 32 Pecs I/II: 18	84 (88%) PVB: 2 Pecs I/II: 81	< 0.001^+^
Intraoperative TIVA, n (%)	23 (8%)	32 (33%)	< 0.001^+^
Intraoperative Ondansetron, *n (%)*	0 mg: 15 (5%) 4 mg: 245 (89%) 8 mg: 14 (5%) 12 mg: 0 (0%)	0 mg: 6 (6%) 4 mg: 88 (92%) 8 mg: 1 (1%) 12 mg: 1 (1%)	0.11^+^
Intraoperative Dexamethasone, *n (%)*	0 mg: 45 (16%) 4 mg: 100(36%) 6 mg: 69 (25%) 8 mg: 49 (18%) 10 mg: 12 (4%)	0 mg: 2 (2%) 4 mg: 22 (23%) 6 mg: 20 (21%) 8 mg: 51 (53%) 10 mg: 1 (1%)	< 0.001^+^

^+^*P*-values calculated from chi-square test; TIVA = total intravenous anesthesia

### Total opioid consumption

The median total perioperative opioid consumption was lower in the ERAS group compared to the Pre group (163.8 mg in the Pre group vs. 111.4 mg oral morphine equivalents in the ERAS group (*p* < 0.001). Opioid consumption decreased in the ERAS group throughout the intraoperative, PACU, and hospital floor settings (Table 3), with the most dramatic in the PACU setting, where ERAS was associated with a decrease from 36.7 mg to 15.4 mg oral morphine equivalents (*p* < 0.001).

**Table 3:** Perioperative Opioid Consumption

Variable Oral morphine equivalents, mg	Pre Group (*n* = 276)	ERAS Group (*n* = 96)	*p*-value
Total Opioid use, *Mean (SD)*	163.8 (73.2)	111.4 (46.0)	< 0.001^*^
Intraop Opioid use, *Mean (SD)*	93.6 (33.8)	73.4 (29.8)	< 0.001^*^
PACU Opioid use, *Mean (SD)*	36.7 (30.8)	15.4 (19.3)	< 0.001^*^
Hospital Floor Opioid use, *Mean (SD)*	33.4 (44.5)	22.6 (19.3)	0.001^*^

*P-values calculated from student’s t-test; *SD* standard deviation, *PACU* post-anesthesia care unit

In a multivariable linear regression model for total opioid consumption adjusting for age, prior opioid use, and surgeon, a 43.4 mg reduction in oral morphine equivalents was independently associated with care in the ERAS pathway (95% confidence interval (CI): 31.0–55.8 mg, *p* < 0.001). A sensitivity analysis to assess the impact of our institution’s transition to a 23-h stay on the total opioid consumption revealed an independent reduction of 13 mg of oral morphine equivalents (95% CI + 0.9 to − 28.3 mg) associated with the transition which was not statistically significant (*p* = 0.065); the effect size of ERAS on opioid dose was slightly attenuated but remained statistically and clinically significant (33.7 mg reduction; 95% CI 17.6–49.8 mg, *p* < 0.001).

### Highest NRS pain score

In the recovery room, the highest pain score was lower in the ERAS group compared to the Pre group (median [IQR]; Pre group 6 [4, 7]; ERAS group 4 [1.75, 6]; *p* < 0.001). On the hospital floor, patients in the ERAS group only reported a 0.5 point decrease in highest pain, though the distribution of scores was on average 1-point lower for patients in the ERAS group (median [IQR]; Pre group: 6 [5, 8]; ERAS group 5.5 [4, 7]; *p* < 0.001).

### Lorazepam use for pectoral muscle spasm

Patients in the ERAS group required less intravenous lorazepam in the PACU compared to the Pre group (18% to 8% (*p* = 0.04)). Furthermore, patients in the ERAS pathway required less lorazepam on the hospital floor, from a mean of 1.64 ± 1.8 mg to 0.87 ± 0.75 mg (*p* < 0.001).

### Incidence of PONV

Total perioperative incidence of PONV significantly decreased from 50% to 27% (p < 0.001), with most of the decrease accounted for in the post-PACU/hospital floor setting from 43% to 7% (p < 0.001). However, use of antiemetics during PACU recovery remained unchanged in the ERAS group (*p* = 0.28) (Table 4).

**Table 4:** Incidence of Postoperative Nausea and Vomiting

Variable	Pre Group (*n* = 276)	ERAS Group (*n* = 96)	*p*-value
Perioperative: any antiemetic, *n (%)*	137 (50%)	27 (28%)	< 0.001^+^
PACU: any antiemetic, *n (%)*	53 (19%)	24 (25%)	0.28^+^
Hospital Floor: any antiemetic, *n (%)*	119 (43%)	7 (7%)	< 0.001^ψ^

^ψ^*P*-values calculated from Fisher’s exact test

^+^*P*-values calculated from chi-square test

*P-values calculated from Mann-Whitney test

*PACU* post-anesthesia care unit

### Surgery times and length of stay

Implementation of the ERAS pathway was associated with a 5-min increase in non-procedural operating room time: 54.8 ± 13 min in ERAS group vs 49.1 ± 12.9 min in Pre group, *p* < 0.001). Total time spent in the PACU decreased by 12 min (mean (SD) 136.3 ± 48.8 min in ERAS group vs 147.5 ± 51.9 min in Pre group, *p* = 0.05). All patients in the ERAS group were discharged on postoperative day 1, while 27 patients in the Pre group were discharged on postoperative days 2–4. The median hospital length of stay in the ERAS group was 1144 min [IQR 992–1259] compared to 1187 min [IQR 1058–1344] in the Pre group (*p* = 0.006), a difference which was not felt to be clinically significant. Furthermore, in a sensitivity analysis adjusting for the impact of the transition to a 23-h stay hospital, the decrease in length of stay independently associated with the ERAS pathway was no longer statistically significant (67 min reduction, 95% CI -233 to + 98 min), *p* = 0.42).

## Discussion

We present the implementation of a multimodal ERAS pathway for total mastectomy in a 23-h stay hospital that led to improved acute post-surgical analgesia and decreased incidence of PONV. Our pathway protocol is consistent with other ERAS pathways, including a recently published ERAS Society endorsed consensus statement for breast reconstruction surgery, in its focus on multimodal pain management and PONV prophylaxis throughout the perioperative period [17]. It differs from other ERAS pathways for breast surgery in its inclusion of Pecs block for postoperative analgesia [11, 12]. This multimodal approach was associated with a 30% reduction in total perioperative opioid consumption, which was independent of a concurrent transition to a 23-h-stay hospital, and a reduction highest NRS pain score in the PACU by 2 points. Collectively, our data suggests that there is a significant improvement in pain control in the PACU with ERAS implementation. We found less improvement in pain control in the post-PACU hospital stay, as evidenced by the modest reduction in opioid consumption and lack of difference in pain scores, which may reflect the limited duration of regional analgesia and difficulty in implementing consistent use of non-opioid analgesics postoperatively.

Our ERAS pathway is novel in its inclusion of Pecs blocks. Pecs blocks have several potential advantages over a paravertebral block. Compared to the paravertebral block, Pecs blocks are more superficial blocks and presumably have a lower risk of pneumothorax. They also do not carry the risk of hypotension from an inadvertent sympathectomy, which can occur with paravertebral blocks [18]. In our institutional experience, the Pecs blocks are easier to learn and achieve proficiency than paravertebral blocks. Furthermore, since the Pecs block is a plane block rather than a nerve block, it can be safely performed in while the patient is under general anesthesia and in the supine position, thereby reducing patient discomfort and anxiety. Implementation of our ERAS pathway with intraoperative Pecs blocks only increased the non-surgical operating room time by 5 min, which has been acceptable to our colleagues and institutional workflow.

A common cause of discomfort after total skin-sparing mastectomy with reconstruction is from raising the pectoralis muscles and the subsequent muscle spasm. Notably, fewer patients in the ERAS group required lorazepam postoperatively, and those who requested lorazepam required a significantly lower dose. This suggests decreased muscle spasm and chest discomfort during recovery, which may be due to the effect of the Pecs block type I on muscle tone in the pectoralis major and minor.

The incidence of PONV following mastectomy has been reported to be as high as 80% [19–21]. However, the incidence of PONV can be reduced to 10–50% with the use of single or combination antiemetics, intraoperative propofol infusion, or limiting volatile anesthetics [19–21]. Therefore, it is not surprising that our ERAS patients had a lower incidence of PONV when compared to the Pre group, since a higher proportion of patients in our ERAS pathway received these interventions. Of note, PONV in the PACU setting remained unchanged in both groups. We suspect that the lack of difference seen in the PACU could be due to active elicitation of such symptoms in the immediate postoperative period with aggressive treatment.

Although many ERAS pathways have resulted in a significantly decreased hospital length of stay [11, 22], our ERAS pathway did not, after adjustment for the institutional transition to a 23-h stay hospital which occurred during the study period. This is not surprising as the majority of patients left on POD#1 prior to ERAS implementation and the hospital transition to a 23-h stay. Thus, in this population, the value of implementing ERAS principles in the limited stay setting lies in the improved quality of care and patient experience by reduction in opioid use and PONV, as well as maximizing the patient’s likelihood of successful discharge. Furthermore, in light of the current opioid epidemic, it is imperative that we continue to embrace opioid-sparing practices, particularly if patient outcomes are similar if not improved in the acute setting.

This study also highlights that the traditional metrics that we use to measure the success of an ERAS pathway of length of stay may not be useful when evaluating the application of ERAS to the outpatient setting. The concept of applying ERAS principles to outpatient surgery is a relatively new one, as most ERAS programs have targeted surgeries with longer length of stays. The current literature that applies ERAS principles to outpatient surgery are essentially proof-of-concept studies with small sample sizes and restrictive inclusion criteria which may limit their widespread applicability [12–14]. Our study adds to the literature in that we applied ERAS principles to our entire population of patients undergoing mastectomy. As ERAS principles are applied to more outpatient surgeries, we believe there must be a shift in how we measure success. Instead of focusing on length of stay, we must instead focus on quality of care metrics, such as patient satisfaction, quality of life measures, post-discharge opioid consumption or development of chronic post-surgical pain.

This study has several limitations. Although providers were aware of the ERAS pathway, the actual interventions were not strictly enforced, and thus patients did not always receive all recommended interventions, as evidenced by a lower compliance in intraoperative TIVA. Furthermore, although the baseline characteristics were the same between patients who underwent surgery before and after implementation of ERAS, this was not a randomized study, and therefore it can be argued that mere awareness of a multimodal pathway targeting pain and PONV can influence the patient’s recovery experience [23].

To assess for PONV we used the surrogate of any antiemetic administered as nausea or vomiting is not specifically recorded in the medical record. The true incidence of PONV would be underestimated if the patient experienced nausea but no antiemetic was given, while it would be overestimated if antiemetics were co-administered with opioids to prevent nausea postoperatively.

Lastly, it is important to note that we are currently unable to measure the efficacy of any individual intervention in the ERAS pathway on postoperative pain or PONV. However, it would have been ethically challenging to randomize and withhold certain interventions with known, evidence-based benefits, especially since our institution supports several other ERAS protocols with similar interventions; thus, this intervention was implemented as a hospital-level quality improvement initiative without randomization and data are reported as such.

Our results are consistent with prior similar studies that introduce multimodal, opioid-sparing interventions, and to date we are not aware of any studies that employ a multimodal pathway including regional anesthesia for total mastectomy. Future improvements to our pathway will include optimizing pre-, post-operative, and post-discharge medications for pain and nausea and taking a more personalized approach for medications such as scopolamine based on risk factors.

## Conclusion

Implementation of an enhanced recovery after surgery pathway for total mastectomy that emphasizes multimodal analgesia and Pecs blocks was associated with a reduction in perioperative opioid consumption and post-operative nausea and vomiting without disrupting the operating room workflow in a 23-h-stay hospital setting. Future studies are needed to determine which individual interventions contribute the most to quality of recovery in the acute perioperative period, as well as the long term effects of ERAS implementation on chronic postsurgical pain and opioid use.

## Additional file

Additional file 1: UCSF Opioid Equivalence Table. (PDF 238 kb)

## Abbreviations

ASA: American Society of Anesthesiology
BMI: Body mass index
EHR: Electronic health record
ERAS: Enhanced recovery after surgery
IQR: Interquartile range
NRS: Numeric rating scale
OR: Operating room
PACU: Post-anesthesia care unit
PONV: Post-operative nausea and vomiting
Pre: Pre-ERAS group
RCT: Randomized control trial
SD: Standard deviation
TIVA: Total intravenous anesthesia
UCSF: University of California, San Francisco
