# Supplementary material for: Improved analgesia and reduced post-operative nausea and vomiting after implementation of an enhanced recovery after surgery (ERAS) pathway for total mastectomy
Source: BMC Anesthesiol. 2018 Apr 16;18:41. doi: 10.1186/s12871-018-0505-9 (PMC5902852; doi:10.1186/s12871-018-0505-9)
Supplement: Supplementary file 1 — UCSF Opioid Equivalence Table. (PDF 238 kb) [file 12871_2018_505_MOESM1_ESM.pdf]

UCSF OPIOID EQUIVALENCE TABLE

The contents of this table are to be used as a guide for approximation of opioid equivalence. This table is not intended for opioid conversion.
Due to inter- and intraindividual differences, these values are approximations.
When calculating equivalence for transdermal opioids or agonist/antagonist class medications, please consider consultation with the Palliative Care, IP3, Acute or Chronic Pain SVC
Healthcare professionals should exercise sound clinical judgment and individualize patient care based upon the patient's condition.

Introduction

The purpose of an opioid equivalence table is to approximate the degree of opioid tolerance a patient demonstrates based on the prescribed opioids they have been consuming for a period of time. The United States Food & Drug Administration (FDA) suggests using a two week period of daily use of an opioid as the minimum time requirement for determining opioid equivalence. These values may also be used to determine opioid requirements needed for a certain outcome (eg NRS <5 or functional improvement such as deep breathing/coughing) and helps track patient progress or regress.

Several opioid conversion calculators exist online or as applications. This table provides evidence-based and expert-based values to approximate opioid equivalence.

How to use this table:

Mr G presents to the hospital for abdominal pain. For the last three months he has been prescribed oxycodone sustained-release (Oxycontin™) 30mg by mouth twice a day (#60 per month) and hydromorphone 4mg by mouth every 4 hours as needed (#180 per month). He states he consumes his OxyContin™ as prescribed. When asked how much hydromorphone he takes, he tells us that he consumes approximately 4 (4mg) pills per day. To calculate how much this equates to in oral morphine equivalents (units described as OME or sometime MEQs):

1. Calculate Daily Use of Each and Every Opioid
Oxycodone 60mg/day
Hydromorphone 16mg/day

2. Calculate equivalence by using the table below
Oxycodone 60mg/day = Morphine 90mg/day
Hydromorphone 16mg/day = Morphine 64mg/day

3. Add the total oral morphine equivalents
Morphine 90mg/day + Morphine 64mg/day = 154mg/day oral morphine equivalents

The FDA has developed the Risk Evaluation and Mitigation Strategy (REMS) program for the prescribing of opioids due to consequences from unsafe practice. At this time enrollment in REMS regarding extended-release/long-acting opioids is voluntary and highly-encouraged.[1] When a patient is on >60 oral morphine equivalents for at least two weeks, a patient is considered "opioid tolerant." It suggests that pain management may be a greater challenge and non-opioid analgesics should be employed.

The purpose of this table is NOT for converting from one opioid to another. The values listed here may overestimate conversion from one opioid to another, therefore for unique opioids, or higher doses, we suggest consultation with one of the Pain Services (Acute Pain SVC, Chronic Pain SVC, IP3) or the Palliative Care, Symptom Management SVCs.

Moffitt-Long Hospital Contact Numbers

UCSF ML Acute Pain	pager: 443-2398
UCSF Chronic Pain	pager: 443-4332
UCSF Palliative Care	pager: 443-4727

Mission Bay Hospital Contact Numbers

UCSF MB Adult Pain	pager: 443-2676 (voalte20450)
UCSF IP3 (Pedi Pain)	pager: 443-6100 (voalte514-9500)
UCSF Symp Mgmt SVC	pager: 443-2767

[1]Extended-Release/Long-Acting (ER/LA) Opioids: http://www.fda.gov/Drugs/DrugSafety/InformationbyDrugClass/ucm163647.htm.

UCSF OPIOID EQUIVALENCE TABLE

The contents of this table are to be used as a guide for approximation of opioid equivalence. This table is not intended for opioid conversion. Due to inter- and intraindividual differences, these values are approximations.
When calculating equivalence for transdermal opioids or agonist/antagonist class medications, please consider consultation with the Palliative Care, IP3, Acute or Chronic Pain SVC
Healthcare professionals should exercise sound clinical judgment and individualize patient care based upon the patient's condition.

UCSF University of California San Francisco

DRUG		ORAL (mg)	PARENTERAL (mg)
**Morphine**		30	10
**Morphine CR/ER (MS Contin)** (Kadian) & (Avinza) are	Non-Formulary	30	-
**Hydrocodone**		30	-
**Hydrocodone ER (Zohydro)(Hyslinga)**	Non-Formulary	30	-
**Oxycodone**		20	10
**Oxycodone CR (Oxycontin)**		20	-
**Hydromorphone (Dilaudid)**		7.5	1.5
**Hydromorphone ER (Exalgo)**	Non-Formulary	7.5	-
**Meperidine (Demerol)***		300	100
**Codeine****		200	100
**Propoxyphene (Darvon)*****	Non-Formulary	200	-
**Oxymorphone (Opana)**	Non-Formulary	10	1
**Oxymorphone ER (Opana ER)**	Non-Formulary	10	-
**Levorphanol**		4	2
**Fentanyl (Sublimaze)**		-	0.1
**Fentanyl TTS (Duragesic)**		See Separate Recommendations	
**Tramadol (Ultram)**		120	100
**Tramadol ER (Ultram ER)**	Non-Formulary	120	-
**Tapentadol (Nucynta)**	Non-Formulary	120	-
**Tapentadol ER (Nucynta ER)**	Non-Formulary	120	-
**Buprenorphine (Subutex)**	Non-Formulary	0.4 (sublingual)	0.3
**Buprenorphine TTS (Butrans)**	Non-Formulary	See Separate Recommendations	
**Nalbuphine (Nubain)**	Non-Formulary	-	10
**Pentazocine (Talwin)**	Non-Formulary	-	30

* *Meperidine's metabolite normeperidine lowers the seizure threshold and is not recommended for analgesia. Meperidine is approved for use for post-op rigors (in PACU) and single-dose administration for short procedures, e.g., colonoscopy.*
** *Codeine has been removed from the Benioff Children's Hospital formulary due to CYP4502D6 variability*
****Propoxyphene-containing compounds (Darvon, Darvocet) were removed from the market in 2011 by the FDA due to increasing safety concerns regarding cardiac toxicity*

- Mixed Agonist/Antagonist Class opioids have a ceiling effect. Consider consultation with a Palliative or Pain SVC.

References

- McPherson ML. Demystifying Opioid Conversion Calculations: A Guide for Effective Dosing. Bethesda, MD: ASHP, 2009.

UCSF OPIOID EQUIVALENCE TABLE

The contents of this table are to be used as a guide for approximation of opioid equivalence. This table is not intended for opioid conversion.
Due to inter- and intraindividual differences, these values are approximations.
When calculating equivalence for transdermal opioids or agonist/antagonist class medications, please consider consultation with the Palliative Care, IP3, Acute or Chronic Pain SVC
Healthcare professionals should exercise sound clinical judgment and individualize patient care based upon the patient's condition.

- Transdermal Opioid Systems have a wide variability in equivalence and caution should be taken in measuring equivalence.

FENTANYL TRANSDERMAL THERAPEUTIC SYSTEMS

**Fentanyl Patch (Duragesic)** (mcg/hr)	**Oral Morphine Equivalents** (mg/day)
**12.5***	NOT FOR INITIATION
**25**	60-134
**50**	135-224
**75**	225-314
**100**	315-404

Please consult the Pain or Palliative Care SVCs regarding initiation or dose adjustments of a transdermal therapeutic system.
Use Apex orderset 1081.
The equivalence guidelines should not be used to convert fentanyl patch to other therapies. Because the calculation to fentanyl is conservative, use of this table to convert to other opioids may overestimate the dose of the new agent.
*May not initiate with this dose. The 12.5 mcg/hr patch is to be used in combination with another fentanyl patch of greater strength

References

www.duragesic.com/prescribing-information.html

BUPRENORPHINE TRANSDERMAL THERAPEUTIC SYSTEMS

**Buprenorphine Patch (Butrans)** (mcg/hr)	**Oral Morphine Equivalents** (mg/day)
**5**	10
**7.5**	15
**10**	20
**15**	30
**20**	40

Non-Formulary

Please note that these doses identify equivalents and SHOULD NOT BE USED FOR CONVERSION.

References

www.butrans.com

METHADONE

Methadone equivalency is not listed. It does not follow linear conversion and has wide inter-individual variability regarding its bioavailability and metabolism. We suggest contacting the Pain or Palliative Care SVCs regarding the calculation of morphine equivalence from methadone.

TRANSMUCOSAL IMMEDIATE-RELEASE FENTANYL (TIRF) PRODUCTS

Transmucosal Immediate-Release Fentanyl (TIRF) products are not listed and consultation with the Acute/Chronic Pain SVC or Palliative Care is suggested. Prescribing TIRF medications requires enrollment in the TIRF-Risk Mitigation and Mitigation Strategy (REMS) program, a FDA-sponsored program. https://www.tirfremsaccess.com/TirfUI/rems/home.action

Version 1.0. Authored by Dr. Ramana Naidu, Dr. Mark Schumacher. Approved by UCSF Pain Committee March 20, 2015
